# Spinal Motion Restriction for Possible Traumatic Cervical Spine Injury: A Scoping Review

**DOI:** 10.7759/cureus.84393

**Published:** 2025-05-19

**Authors:** Jorien Laermans, Eunice M Singletary, Finlay Macneil, Frances Williamson, Tine D'aes, Diana Carmen Cimpoesu, Therese Djarv, Emmy De Buck

**Affiliations:** 1 Centre for Evidence-Based Practice, Belgian Red Cross-Flanders, Mechelen, BEL; 2 Leuven Institute for Healthcare Policy, Department of Public Health and Primary Care, KU Leuven, Leuven, BEL; 3 Cochrane First Aid, Belgian Red Cross-Flanders, Mechelen, BEL; 4 Emergency Medicine, University of Virginia, Charlottesville, USA; 5 First Aid, Australia and New Zealand Committee on Resuscitation, Gosford, AUS; 6 Trauma Service, Emergency and Trauma Centre, Royal Brisbane and Women's Hospital, Brisbane, AUS; 7 Emergency and Prehospital EMS Serviciul Mobil de Urgență, Reanimare și Descarcerare (SMURD), Emergency County Hospital “Sf. Spiridon”, Iasi, ROU; 8 Emergency Medicine, Karolinska Institute, Stockholm, SWE

**Keywords:** cervical collar, cervical spine, prehospital care, scoping review, spinal immobilization, spinal motion restriction

## Abstract

Spinal motion restriction protocols are used worldwide by Emergency Medical Service professionals for patients with suspected spinal injuries. Similar guidance for trained and untrained first aid providers is currently lacking and continuously debated, fueled by ongoing controversy on the use of cervical collars by lay first aid providers and evidence of potential harm of spinal motion restriction practices and devices. A systematic collection of the available evidence may provide much-needed clarity and inform policy.

The aim of this review was to scope the literature published since 1999 on the effectiveness of prehospital cervical spinal motion restriction, as part of the continuous evidence evaluation process of the International Liaison Committee on Resuscitation. In this scoping review, spinal motion restriction is defined as attempting to maintain the spine in anatomic alignment and minimizing gross movement with or without adjuncts or devices.

We searched MEDLINE, Embase, and CINAHL Plus from inception until July 30, 2024, for studies comparing spinal motion restriction with no spinal motion restriction or with other types of spinal motion restriction. Extracted data were synthesized narratively using frequency counting and visualized in interactive evidence gap maps.

Sixty-six studies were included. The vast majority (76%) of the 47 experimental studies were performed on live human volunteers to assess a range of cervical motion and adverse effects. The 19 observational studies mainly investigated the risk of secondary spinal injury, functional outcomes, and adverse effects in trauma patients. In none of the included studies, spinal motion restriction interventions were applied by first aid providers (whether trained or untrained). Only seven studies were conducted in low- and middle-income countries. Just two studies investigated improvised devices, which may be useful for first aid providers in low-resource settings.

This scoping review provides a comprehensive and graphic overview of the available evidence on prehospital cervical spinal motion restriction. In doing so, it exposed multiple research gaps, including the lack of studies in which these interventions are applied by first aid providers, the paucity of research performed in low- and middle-income countries, and a further need for research involving adults and children with possible traumatic cervical spine injuries, as well as studies using improvised devices. This scoping review may serve as a basis for future systematic reviews that are required to confidently formulate evidence-based first aid treatment recommendations. It could also contribute to minimizing research waste and help prioritizing primary research on this topic.

## Introduction and background

For multiple decades, full spinal immobilization was a routine practice in the acute management of patients with actual or potential traumatic spinal injuries, traditionally involving the application of a rigid or semi-rigid cervical collar and a long spine board with head blocks or straps [1]. The rationale for immobilizing the spine during extrication and transportation was to prevent the worsening of existing injuries or the creation of new injuries. However, spinal injuries are relatively uncommon, reported to be occurring in as little as 1%-7% of non-penetrating trauma patients [2-4]. Additionally, a growing evidence base suggests that these immobilization practices can delay extrication and transportation, hinder airway management, increase intracranial pressure, compromise respiratory status, lead to pressure ulcers, and cause overall patient discomfort [5-8]. As a consequence, a widespread de-adoption has occurred, with Emergency Medical Service protocols eliminating or limiting the use of the long spine board and terminology shifting from “spinal immobilization” to “spinal motion restriction” to highlight these changes [1].

Similar guidance for first aid providers continues to be debated and is of ongoing concern to organizations that provide first aid training and services to the public and/or those with a duty to respond (e.g., lifeguards, police, search and rescue, and ski patrols). Therefore, the First Aid Task Force of the International Liaison Committee on Resuscitation (ILCOR) has repeatedly prioritized this topic as part of its continuous evidence evaluation process.

During a 2015 ILCOR systematic review that focused on mechanical cervical immobilization devices accessible to first aid providers (i.e., cervical collars and sandbags with tape), very low-certainty evidence was identified showing an increased intracranial pressure with cervical collar application [9,10]. This evidence was integrated into the first aid guidelines of many ILCOR member organizations, with the 2015 First Aid Guidelines of the European Resuscitation Council (ERC) [11] and the American Heart Association and American Red Cross (AHA/ARC) [12] stating that the routine application of a cervical collar by a first aid provider is not recommended. Instead, manual support of the head in a position limiting movement until experienced healthcare providers are available was recommended.

In 2019, ILCOR performed two scoping reviews: one to search for additional publications on cervical spinal motion restriction (this time also including long spine boards) [13] and another one on the effectiveness of manual stabilization techniques such as the trap-squeeze and head-squeeze techniques [14]. Given the limited amount of (additional) evidence and discussions around the ability of first aid providers to properly apply a cervical collar and the non-routine use of cervical collars among others, a new systematic review or reconsideration of recommendations for practice was not considered relevant. The 2021 ERC First Aid Guidelines, therefore, remained unchanged from 2015 [15].

The current scoping review was conducted in 2024 by the ILCOR First Aid Task Force using a wide approach that encompassed the previous work done in 2015 and 2019. The aim was to identify the literature published since 1999 on cervical spinal motion restriction performed by (trained) first aid providers, to get a broad overview of the scientific research on the effectiveness and the changes in practice pertaining to spinal motion restriction. As accepted terminology remains debated, for this review, spinal motion restriction was defined as “attempting to maintain the spine in anatomic alignment and minimizing gross movement, with or without adjuncts or devices.”

## Review

Methods

Although no protocol was developed, we adhered to the methodological standards of ILCOR Task Force Scoping Reviews [16]. Reporting was done in accordance with the Preferred Reporting Items for Systematic Reviews and Meta-analyses Extension for Scoping Reviews (PRISMA-ScR) checklist [17].

Eligibility Criteria

All identified records were screened against the inclusion and exclusion criteria shown in Table 1. Previously published systematic and scoping reviews on this topic led us to expect a very limited evidence base on prehospital cervical spinal motion restriction applied by first aid providers (trained or untrained). Therefore, we decided to apply broader eligibility criteria and include studies that would provide indirect evidence, for example, experimental studies in healthy volunteers and cadavers.

**Table 1:** Eligibility criteria PICOST: population, intervention, control, outcomes, study design, and timeframe

PICOST item	Included	Excluded
Population	Adults or children with possible cervical spine injury due to non-penetrating trauma	Adults or children with penetrating trauma
	Studies in healthy volunteers	-
	Studies in human cadavers	-
Intervention	Studies were included if they met all of the following criteria (A, B, and C):	-
	(A) Motion restriction was performed at the level of the cervical spine, with or without restriction at the level of the lower (i.e., thoracic, lumbar, and sacral) spine	Studies that did not concern motion restriction at the level of the cervical spine (e.g., thoracic spinal motion restriction)
	(B) Devices used for motion restriction are commonly and readily available to (trained) first aid providers. Discussions within the Task Force revealed that the types of devices that first aid providers are trained to use vary largely between countries and between settings within countries, on top of the varying availability of those devices themselves. For instance, in the US, ski patrollers are trained in the use of a long backboard and many use vacuum mattresses. As a result, a wide range of devices was considered relevant for inclusion in this scoping review: any type of improvised or commercially available cervical collar (soft, semi-rigid, or rigid), long backboard, head blocks, straps, vacuum mattress, and scoop stretcher	Studies using devices that are not commonly and readily available to (trained) first aid providers, such as the Kendrick extrication device, the Pediatric Immobilization and Extrication System (SIPE) Baby Rescuer device, the halo vest, and the Minerva jacket
	(C) Techniques used for motion restriction do not require extensive amounts of specialized training	-
	Extrication studies were included if they allowed evaluation of the effect of a technique/device for spinal motion restriction and if this technique/device was relevant to first aid. As such, the following comparisons were included, and analyzed separately: uninstructed self-extrication with no cervical collar versus uninstructed self-extrication with a cervical collar; uninstructed self-extrication (with or without a cervical collar) versus instructed self-extrication (with or without a cervical collar)	-
	Studies assessing the impact of implementing a more restrictive spinal motion restriction protocol were also included and analyzed separately	-
		Studies looking at the effect of lifts, transfers, and/or carries in combination with cervical spinal motion restriction, as they provide information on the effectiveness of the method of the lifting/transfer/carry techniques, rather than on the effectiveness of the restriction method
Comparator	No cervical spinal motion restriction	Other comparators
	Another type of cervical spinal motion restriction	
Outcome	Any clinical outcome related to the patient, including the incidence of (secondary) spinal injury; cervical (range of) motion; functional outcomes and/or survival; length of hospital stay; adverse effects of spinal motion restriction occurring within 24 hours after the injury (i.e., on the way to the emergency department or inside the emergency department), including effects on intracranial, cerebrospinal fluid, or cerebral perfusion pressure; respiratory function; cardiovascular function; and pain and discomfort	Adverse effects of spinal motion restriction that were delayed or occurred after admission to a hospital associated with prolonged immobilization (e.g., skin breakdown, formation of pressure ulcers after days in the Intensive Care Unit or hospital ward)
		Studies reviewing the effect of spinal motion restriction on the ease of intubation with different airway devices or laryngoscopes, or on the ease of performing vertebroplasty, laminectomy, or other surgical interventions
Study design	Randomized controlled trials (RCTs); non-RCTs; interrupted time series, controlled before-and-after studies, cohort studies, case series	Case reports, studies performing a single measurement (e.g., feasibility study, proof-of-concept study), gray literature, social media, non-peer-reviewed studies, unpublished studies, conference abstracts, and trial protocols (as there was abundant evidence from published studies)
Timeframe	1999-2024	<1999

Search Strategy

A search strategy was developed with assistance from Mark S. McKone, an information specialist at the Wake Forest School of Medicine Coy C. Carpenter Library in Winston-Salem, NC, USA (see the appendix). Articles for review were obtained by searching the following databases, with final searches conducted on July 31, 2024: Ovid MEDLINE(R) ALL <1946 to July 31, 2024>, Embase <1974 to 2024 July 30>, and CINAHL Plus with Full text (1936 - Present) via EBSCOhost.

Additional studies were identified by screening the reference lists of the included studies and by screening the included studies of systematic reviews and scoping reviews retrieved via the database searches and reference list screening. Finally, the members of the ILCOR First Aid Task Force were asked to review the list and provide additional studies for review to ensure there were no obvious omissions.

Study Selection

Records from the database searches were downloaded and imported into Covidence [18] for the removal of duplicates and screening. Two reviewers (JL and ThD) independently screened the titles and abstracts and then evaluated the full texts for relevance. Any discrepancies were discussed, and if no consensus could be reached, a third reviewer was consulted (EMS).

Data Charting, Synthesis, and Presentation

Data extraction from the included studies was done by a single reviewer (JL/EMS/FM/FW/DC/ThD/collaborator GF) and checked by a second reviewer (TiD/JL). For each study, the following data were extracted and charted: year of study, country, study design, population characteristics, first aid intervention characteristics, and quantitative outcomes. Reported equity-related characteristics were gathered using the PROGRESS-Plus framework items [19,20]. Extracted information was synthesized narratively using frequency counting. Studies were classified into four main categories: (1) spinal motion restriction vs. no spinal motion restriction, (2) mutual comparisons of types of spinal motion restriction, (3) extrication studies, and (4) post- vs. pre-implementation of spinal motion restriction protocols. Data concerning main categories 1 and 2 were coded in EPPI-Reviewer [21] by a single reviewer (JL) and checked by a second reviewer (TiD) and subsequently visualized in interactive evidence gap maps using EPPI-Mapper [22].

Results

Study Selection

**Figure 1:**
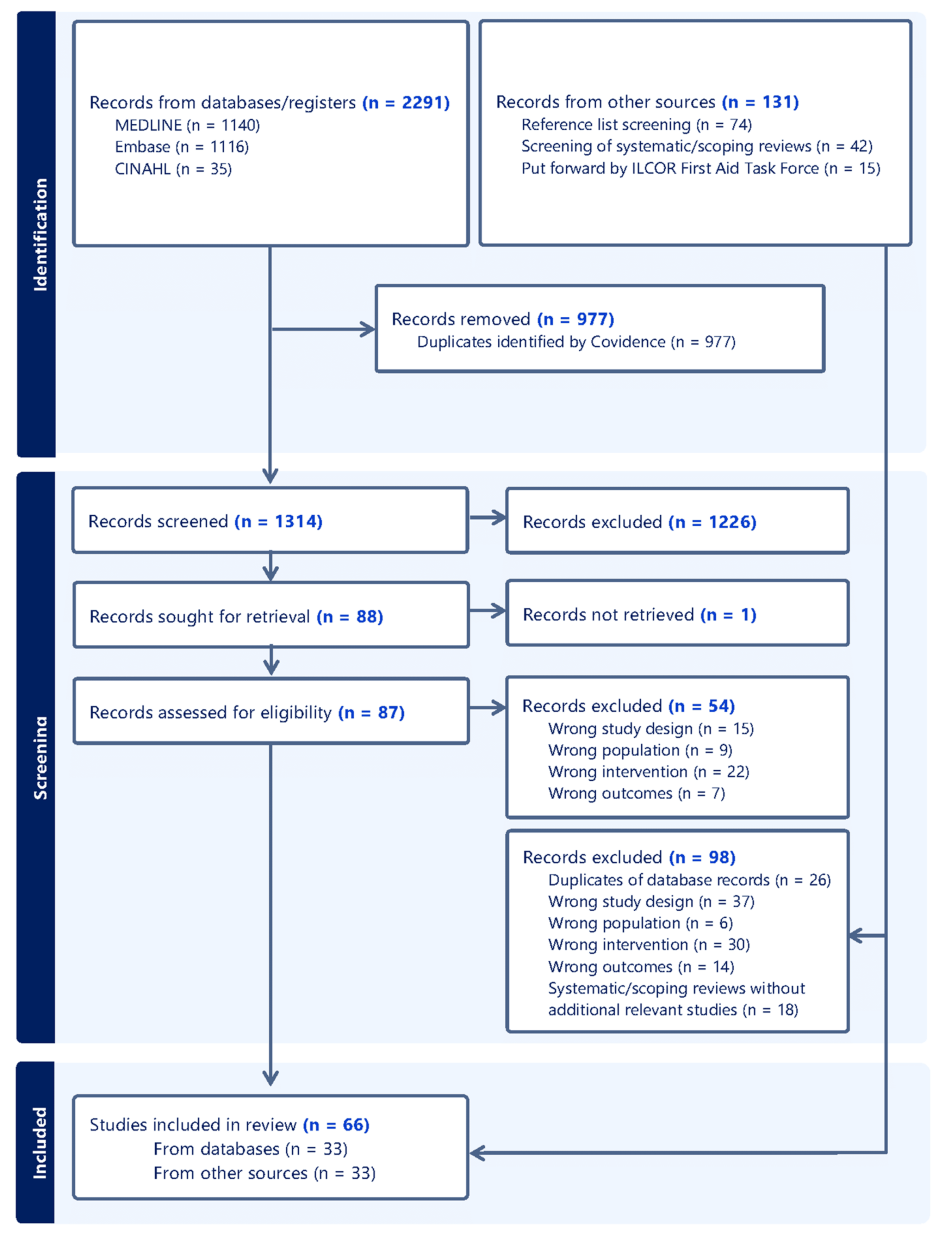
PRISMA flow chart PRISMA: Preferred Reporting Items for Systematic Reviews and Meta-analyses

A total of 2,291 records were retrieved, and after removing 977 duplicates, 1,314 records remained for the title and abstract screening (see Figure 1 for the PRISMA flow chart). After excluding 1,226 records, a total of 87 full texts were screened. After screening the reference lists of all included studies, looking at existing systematic and scoping reviews [5-8,23-39], and asking for input from the ILCOR First Aid Task Force, 131 additional records were reviewed, and 33 relevant studies were identified. Taken together, 66 studies [40-105] were included in this scoping review.

Study Characteristics

In the following paragraphs, the study characteristics are described based on frequency counting across all 66 included studies. For the sake of manuscript readability, references are not provided in-text but are listed in Table 2. A detailed overview of all the extracted data per study can be found in Appendix 4 on the ILCOR CoSTR website [106].

**Table 2:** Overall study characteristics *Patients presenting at the emergency department for a lumbar puncture to rule out meningitis or a subarachnoid bleed

Study characteristic	Value	References
Geographical location	USA	[44,46,49,51,52,55,61,62,66,67,73,78,80,83,86,88,90,92-94,96-98,102,103,105]
	UK	[53,59,60,68,84,91,101]
	The Netherlands	[56,58,72,77,85,100]
	Australia	[41,45,81,82]
	Canada	[43,79,87]
	Turkey	[42,50,104]
	Taiwan	[48,71,75]
	Poland	[69,70,95]
	Germany	[74,99]
	Iran	[40,76]
	Italy	[54]
	Spain	[57]
	Iceland	[64]
	Korea	[65]
	14 Asian countries (PATOS registry)	[47,63]
	Not reported	[89]
Study design	RCT	[40,52,54,55,61,64,65,69,70,72,76,77,86,88,91,95-98,101,103,105]
	Non-RCT	[42-44,48,51,53,57-60,67,74,80,84,87,89,90,92,99]
	Cohort study	[46,47,49,63,73,79,81,100]
	Interrupted time series	[50,68,102]
	Case series	[41,45,56,66,78,82,93]
	Retrospective chart reviews	[62,71,75,83,85,94,104]
Study population	Healthy volunteers	[40,42,48,51-55,57-59,61,64,65,67-70,72,77,78,80,84,86-88,91-93,95-99,101-103,105]
	Trauma patients	[41,45-47,49,50,56,60,62,63,71,73,75,76,79,81-83,85,94,100,104]
	Cadavers	[43,44,74,89,90]
	Other*	[66]
Interventions	Cervical collar only, as the sole intervention of interest	[23,41,43-45,48,50-55,57,59-61,64-66,68-72,74,75,77,80-82,84,86,89,91-93,95-97,101-105]
	Cervical collar only, as one of several interventions of interest	[58,79,85,87,90]
	Long spine board + cervical collar (with or without head blocks or straps)	[42,58,67,76,78,79,85,87,88,98,99]
	Vacuum mattress + cervical collar (with or without head blocks or straps)	[76,85,88,98,99]
	Long spine board (with or without head blocks or straps)	[58,83,85,99]
	Scoop stretcher + cervical collar	[67]
	Cervical collar + head blocks	[56]
	Unclear - "cervical collar and/or long spine board”	[73]
	Unclear - “neck collar and/or backboard or scoop stretcher”	[47]
	Unclear - "rigid collar, straps/head blocks, etc.”	[62]
	Unclear - "cervical spinal immobilization”	[63]
	Unclear - "spinal motion restriction (vs. spinal immobilization)”	[46,49]
	Unclear - "selective spinal immobilization protocol”	[94]
	Unclear - "preventive spinal immobilization protocol”	[100]
Person applying the intervention	Emergency Medical Services	Observational studies: [23,46,47,49,62,63,73,75,79,81,83,85,94,100]; experimental studies: [52,88]
	Trained/supervised study investigator(s)	Experimental: [59,72,84,92,97]
	Study investigator(s) (no mention of training/supervision)	Experimental: [67,91]
	Paramedic	Observational: [45]; experimental: [87]
	Emergency Medical Technician	Experimental: [51,76]
	Emergency physician	Experimental: [74,80]
	Intensive Care Unit personnel	Experimental: [60]
	Emergency department personnel	Experimental: [66]
	Physiotherapist	Experimental: [53]
	Orthotist	Experimental: [55,64]
	Athletic trainer	Experimental: [61]
	Surgeon	Experimental: [89,90]
	Not specified - "prehospital”	Observational: [50,56,71,104]
	Not specified, but probably study investigator(s)	Experimental: [40,42-44,48,57,68,69,78,86,93,95,98,99,102]
Outcomes of interest	Cervical range of motion	[43,44,48,51-55,57,58,61,64,65,67,74,76,79,80,84,86-92,96,97,99,101,105]
	Adverse effects - pain/discomfort	[45,56,64,67,70,72,76,78,81,85,86,91,92,98,101]
	Adverse effects - intracranial pressure	[50,60,64,66,68,69,74,77,82,93,95,102-104]
	Adverse effects - skin function	[72,91,101]
	Adverse effects - interface pressure	[56,70,72,91,96,97,101]
	Adverse effects - respiratory function	[40,42,85,98]
	Risk of (secondary) spinal cord injury	[41,46,49,73,75,81,83]
	Functional outcomes	[45,47,63,71,94,100]
	Survival	[62,63,71,83,100]
	Length of hospital stay	[62,75,83]

Forty percent (n = 26) of the included studies were conducted in the USA, followed by the UK (n = 7), the Netherlands (n = 6), and Australia (n = 4). Other countries with multiple studies were Canada (n = 3), Turkey (n = 3), Taiwan (n = 3), Poland (n = 3), Germany (n = 2), and Iran (n = 2). Two studies used the Pan-Asia Trauma Outcomes Study (PATOS) multicenter trauma registry from 14 Asian countries.

The evidence base consists of 47 experimental and 19 observational studies: 22 randomized controlled trials (RCTs), 19 non-RCTs, eight cohorts, three interrupted time series, seven case series, and seven retrospective chart reviews. Thirty-eight studies were performed on healthy volunteers, whereas 22 studies involved trauma patients and five used cadavers. Healthy volunteer studies mostly used younger populations, often recruited from university, medical training, or residency programs. In contrast, the studies in trauma patients provided a more balanced image across age categories. Gender/sex distribution was relatively balanced across all studies. A full overview of the reported equity-related data can be found in Appendix 6 on the ILCOR CoSTR website [106].

In 44 studies, cervical collars were the sole device of interest. Five other studies also looked at cervical collar use only, but as one of the multiple interventions of interest. Other interventions studied included a long spine board in combination with a cervical collar (with or without head blocks or straps) (n = 11), a vacuum mattress in combination with a cervical collar (with or without head blocks or straps) (n = 5), a long spine board (with or without head blocks or straps) (n = 4), a scoop stretcher with a cervical collar (n = 1), and a cervical collar with head blocks (n = 1). Three observational studies did not allow the determination of the specific individual interventions used, as they described spinal motion restriction as “having a cervical collar and/or being secured to a rigid spine board” [73], “using a neck collar and/or backboard or scoop stretcher” [47], or “patients could have had a rigid collar, straps/head blocks, etc.” [62]. Five other studies simply referred to “cervical spinal immobilization” [63], “spinal motion restriction vs. spinal immobilization” [46,49], “selective spinal immobilization” [94], or “preventive spinal immobilization” [100] protocols.

In 15 observational studies, the population was restricted to trauma patients receiving prehospital spinal motion restriction interventions from Emergency Medical Services (n = 14) or paramedics (n = 1). In four other observational studies, the devices (either a collar or a collar together with head blocks) were applied “rehospital” with no further information on who applied them. Twenty-four experimental studies explicitly mentioned who applied the intervention, the most frequent being trained/supervised study investigators (n = 5), Emergency Medical Services personnel (n = 2), Emergency Medical Technicians (n = 2), orthotists (n = 2), surgeons (n = 2), and emergency physicians (n = 2). In 15 other experimental studies, although unspecified, it is likely that the interventions were applied by the study investigators themselves, who were often affiliated with the University Hospital’s Departments of Emergency Medicine, Emergency Medical Services, Orthopedics, and (Trauma &) Orthopedic Surgery.

The most frequently studied outcome was cervical range of motion (n = 31). Adverse effects of devices were extensively studied, including pain/discomfort (n = 15), intracranial pressure (n = 14), effects occurring at the device-skin barrier (i.e., skin function (n = 3) and interface pressure (n = 7)), and respiratory function (n = 4). Other outcomes included the risk of (secondary) spinal cord injury (n = 7), functional outcomes (n = 6), survival (n = 5), and length of hospital stay (n = 3).

In the following paragraphs, we provide a synthesized overview of the evidence base per intervention category (with in-text references). For individual and synthesized study results, the reader is referred to Appendix 7 on the ILCOR CoSTR website [106].

Category 1: Spinal Motion Restriction Versus No Spinal Motion Restriction

The characteristics of the 47 studies in this category [40-45,47,48,50,51,53,55,58-60,62-66,68-71,74,75,82,86-89,91-98,102-105] can be accessed interactively here: https://cebap.org/storage/cebap/laermans-2025-egm1.html

**Figure 2:**
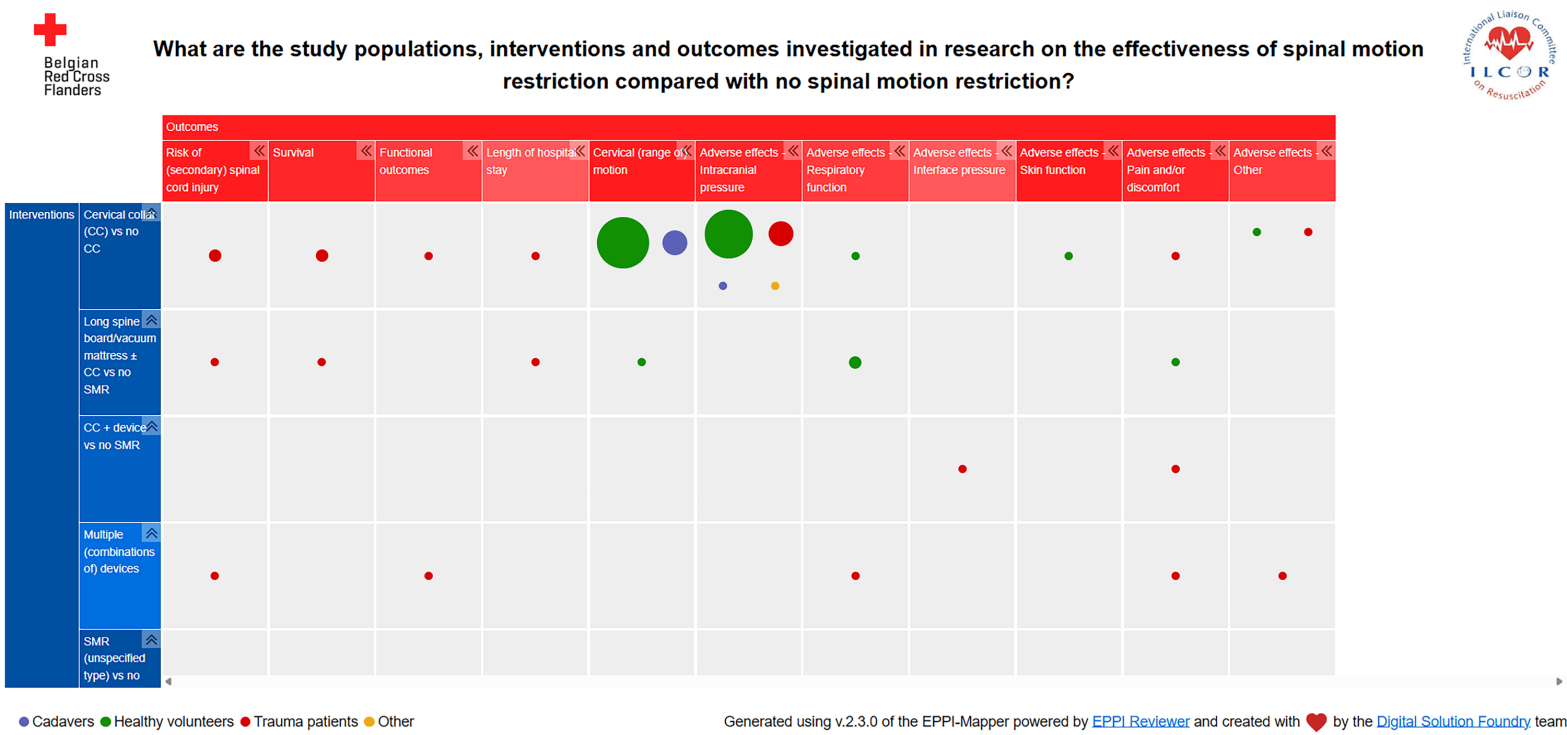
Static version of the evidence gap map for the comparison of spinal motion restriction compared with no spinal motion restriction Different comparisons of interventions are presented as rows, and outcomes are presented as columns. Bubbles denote the existence of one or more studies examining this particular outcome for this particular comparison; the size of the bubble is proportional to the number of studies. Bubbles of different colors indicate different study populations (cadavers, healthy volunteers, trauma patients, and others). Generated using v.2.3.0 of EPPI-Mapper powered by EPPI Reviewer and created by the Digital Solution Foundry team

In this evidence gap map, different comparisons of interventions are presented as rows, and outcomes are presented as columns. Bubbles denote the existence of one or more studies examining this particular outcome for this particular comparison; the size of the bubble is proportional to the number of studies. Bubbles of different colors indicate different study populations (cadavers, healthy volunteers, trauma patients, and others). Lists of studies within each category can be obtained by clicking on the map, and studies can be filtered based on the study design, study population, location, publication date, and publication language. A static overview of this gap map is included in this article as Figure 2.

Thirty-five studies compared cervical collar use with no cervical collar use [40,41,43-45,48,50,51,53,55,58-60,64-66,68,70,71,74,75,77,82,88,89,91-93,95-97,102-105]. Cervical range of motion was investigated in 12 studies that involved healthy volunteers [48,51,53,55,58,64,65,88,92,96,97,105] and in four cadaver studies [43,44,74,89]. The second most frequent outcome concerned an important potential harm of cervical collars, i.e., increased intracranial pressure. This was measured by eight studies involving healthy volunteers [64,68,69,77,93,95,102,103], four studies in trauma patients [50,60,82,104], one cadaver study [74], and one study in patients presenting at the emergency department for a lumbar puncture (to rule out meningitis or a subarachnoid bleed) [66]. The risk of (secondary) spinal injury was examined by three studies in trauma patients [41,45,75].

Five studies [42,78,83,87,98] looked at the effect of using a long spine board or vacuum mattress with or without a cervical collar. Four of those used healthy volunteers to examine cervical range of motion [87], respiratory function [42,98], and pain and/or discomfort [78,98].

Three studies compared an unspecified type of spinal motion restriction with no spinal motion restriction [62,63,94]. In Jao et al. [62] and Jung et al. [63], the association between prehospital cervical spine immobilization (techniques and devices not specified) and mortality and functional outcomes in trauma patients was studied. Stroh and Braude [94] reported on the influence of violations of cervical spinal immobilization protocols (i.e., people who were supposed to be immobilized were not) on patient outcomes.

Category 2: Mutual Comparisons of Several Types of Spinal Motion Restriction

The characteristics of the 32 studies [40,41,43,51,53,55,58,61,64,65,67,69,70,72,76,79-81,86-92,95-99,101,105] in this category can be accessed interactively here: https://cebap.org/storage/cebap/laermans-2025-egm2.html

Twenty-three studies provided evidence on the comparative effectiveness of different types of cervical collars [40,41,43,51,53,55,61,64,65,69,70,72,80,81,86,89,91,92,95-97,101,105]. Five of those studies compared a soft foam collar with a (semi-)rigid collar, examining the effects on cervical range of motion [43,51,89], the risk of (secondary) spinal injury [41,81], and the risk of developing pressure ulcers [81] and hospital-acquired pneumonia [81]. One study compared an improvised collar with a commercially available collar, looking at the effects on cervical range of motion [86].

Another five studies compared one-piece rigid to two-piece rigid cervical collars and investigated the effects on cervical range of motion [61,64,101] and adverse effects [64,72,91,101]. This comparison was of interest to the scoping review team, as in an emergency or out-of-hospital setting, a one-piece collar is often considered to be easier, more efficient, and quicker to apply.

Fourteen studies looked at other mutual comparisons of cervical collars, investigating cervical range of motion [53,55,65,80,86,91,92,96,97,105] and adverse effects [40,69,70,86,92,95-97]. Three studies compared the effect of a cervical collar to another type of device on cervical range of motion. Eisner et al. [51] used a folded towel wrapped around the neck and crossed around the chest, whereas Holla [58] applied a spine board and head blocks, and Roebke [90] used a spray-on foam splint.

Three studies investigated the effects of the combined use of multiple devices on cervical range of motion [79,87,88]. All three compared the use of a cervical collar together with other devices (long spine board with or without head blocks, vacuum mattress with or without head blocks) to the use of a cervical collar only. One of these studies [88] also compared, on the one hand, a long spine board with a cervical collar and head blocks and, on the other hand, a vacuum mattress with a cervical collar and head blocks.

Another three studies compared the combined use of a cervical collar and a certain device (e.g., a long spine board) to the combined use of a cervical collar and another device (e.g., a vacuum mattress), investigating the effects on cervical range of motion [67,76,99] and pain/discomfort [67,76]. One of these studies also compared the cervical range of motion using a long spine board with spider straps to a vacuum mattress with or without head blocks [99].

Category 3: Extrication Studies

The effect of spinal motion restriction methods/devices on the cervical range of motion during simulated extrication from vehicles was assessed in four healthy volunteer studies [52,54,57,84]. Gabrieli et al. [54], Hontoria Hernández et al. [57], and Nutbeam et al. [84] focused on self-extrication from the vehicle, with Hontoria Hernández examining the effect of wearing a cervical collar, whereas Gabrieli et al. and Nutbeam et al. also investigated the effect of receiving instructions for self-extrication. Engsberg et al. [52], on the other hand, compared unassisted self-extrication with or without a cervical collar in place to being assisted by Emergency Medical Service providers to exit the vehicle with a cervical collar in place.

Category 4: Post- vs. Pre-implementation of Selective Spinal Motion Restriction Protocols

Three retrospective cohort studies [46,49,100] investigated the incidence of spinal cord injuries or functional outcomes of trauma patients before and after the implementation of selective spinal motion restriction protocols. Castro-Marin et al. [46] and Clemency et al. [49] both assessed the influence of implementing USA statewide protocols where the least restrictive immobilization techniques were applied only to those patients with significant risk factors (e.g., high-risk mechanism of injury and age > 65 years), abnormal findings on examination (e.g., altered level of consciousness, neurological symptoms such as numbness/tingling/weakness, and complaints of neck pain/tenderness) or an unreliable physical exam (e.g., due to a distracting injury). The newly implemented protocol evaluated by van de Breevaart et al. [100] in the Netherlands was very similar but directed toward preventive spinal immobilization for “every mechanism with risk of vertebral injury” instead of high-risk mechanisms of injury.

Discussion

The current scoping review mapped 66 studies published since 1999 on the effectiveness of spinal motion restriction. Although numerous scoping reviews and systematic reviews have been published on this topic [5-8,23-39], these previous reviews either applied more narrow selection criteria (e.g., only looked at cervical collars or only included RCTs) or were published more than five years ago. To our knowledge, this is the most comprehensive scoping review available.

While reviewing the extensive evidence base, the ILCOR First Aid Task Force noted an important evidence gap: there were no studies that evaluated the benefits and harms of spinal motion restriction where the interventions were applied by first aid providers, either untrained or trained. In addition, the preponderance of the evidence identified came from experimental studies in healthy young adult human volunteers (i.e., with no neck injury or pain) or in human cadaver models, and as such, results may not be generalizable to the population of adults and children with possible traumatic cervical spine injury. Extrapolation of findings from studies using healthy uninjured volunteers to those with injuries may not be valid, due to the absence of muscle spasms in conscious volunteers and the absence of ligament/bony disruption. Furthermore, only seven of the included studies were conducted in low- and middle-income countries [40,42,47,50,63,76,104], which may further hinder the global generalizability of the findings.

The reporting of equity-relevant characteristics was limited, with most studies only providing data on the gender/sex and the age of the participants. Future studies should report these data more thoroughly, considering that potential differences in effects across these factors may be important to inform future decision- and policy-making, in order to enhance health equity.

Multiple studies evaluated the use of a cervical collar with or without adjunct devices. Only two studies looked at improvised devices for spinal motion restriction, which may be useful for first aid providers in low-resource settings, indicating another important evidence gap. One study used a folded fleece jacket collar [86], whereas the other used a folded towel wrapped around the neck and crossed around the chest [51]. In contrast, a vast number of experimental studies included direct comparisons of multiple commercially available cervical collars. Partly because of this, there was marked heterogeneity between studies based solely on the different brands of cervical collars, their design (one-piece or two-piece), and structure (rigid, semi-rigid, soft, or improvised). This heterogeneity might hinder future systematic review teams from performing relevant evidence synthesis, such as meta-analysis. Also, as nearly all studies using a Philadelphia^®^ collar failed to clearly report on the features of the collar (e.g., specific type, design, structure, and manufacturer), categorizing them correctly will require systematic review teams to contact the authors of the individual studies. This categorization is also necessary to have a more trustworthy overview of the evidence on the comparative effectiveness of one-piece vs. two-piece collars.

There are several limitations to this scoping review. We initially did not intend to look for evidence on the effectiveness of implementing selective spinal motion restriction protocols, and therefore, our search strategy was not sufficiently tailored to identify this type of study. Future systematic review teams should consider searching for these studies, mapping the available evidence to identify potential research gaps, and synthesizing the available data. This is especially important to determine if protocol-based selective spinal motion restriction should be applied by trained first aiders in high-risk situations (e.g., older adults, intoxicated people, and high-impact trauma). Similarly, it may be worthwhile to further collect evidence on the effectiveness of different methods or devices for extrication from vehicles, given the high burden of road traffic injuries. Another limitation is the use of (arbitrary) categories that were created after data extraction. Finally, as this was a scoping review, we did not perform formal data synthesis (using, for example, meta-analyses) and did not assess the risk of bias in the individual studies or the certainty of evidence. For this reason, we did not include individual or synthesized study results in this manuscript. Further systematic reviews that apply formal data synthesis, risk of bias, and certainty of the evidence assessments are required to confidently withdraw or formulate recommendations for practice that may influence policy on which practices to apply and which devices to carry. Systematic review teams should certainly consider the usefulness and potential impact of including evidence from healthy uninjured volunteers.

The strengths of the current scoping review lie in its rigorous and systematic methodology, as well as the breadth of the covered topic. In addition, comprehensive, accessible, and interactive visual overviews are provided in the form of evidence gap maps. This review, therefore, serves as a solid foundation for future systematic reviews on one or more narrowly defined research questions that should further inform practice. Furthermore, this review may help reduce research waste and assist with prioritizing primary research on this topic.

## Conclusions

This scoping review mapped 66 studies that contain evidence on the effectiveness of prehospital spinal motion restriction and provides comprehensive and interactive graphic overviews of the evidence base. Most studies identified (47/66) were experimental in design and used healthy volunteers or human cadavers to evaluate the cervical range of motion and potential adverse effects of motion restriction. Only one-third of the studies evaluated cervical spinal motion restriction applied to actual trauma patients, primarily examining the risk of secondary spinal injury, functional outcomes, and adverse effects.

Our review highlights the need for further research involving adults and children with possible traumatic cervical spine injuries, rather than relying on surrogate models. Moreover, it shows that there is a paucity of studies performed in low- and middle-income countries and those investigating improvised spinal motion restriction devices. Finally, no studies were found in which spinal motion restriction was applied by first aid providers, whether trained or untrained. Beyond helping to prioritize primary research, this review serves as a basis for upcoming systematic reviews. These are essential for developing evidence-based treatment recommendations that could shape policies on which practices to adopt, which devices to carry, and how to limit the unnecessary use of cervical spinal motion restriction by first aid and other prehospital care providers.

## Appendices

**Table 3:** Search strategy

Database	Search date	Set	Search string	Explanatory notes	Set results
Ovid MEDLINE(R) ALL <1946 to July 30, 2024>	April 30, 2023	1	exp "Neck Injuries"/ or exp "Cervical Vertebrae"/in		16,191
		2	(exp "Neck"/ or exp "Cervical Vertebrae"/) and (injury or injuries or trauma* or compression).ti,ab,kf,kw.		16,315
		3	(((neck or cervical) adj2 (injury or injuries or trauma* or compression or cadaver* or volunteer*)) or whiplash*).ti,ab,kf,kw.		14,428
		4	("Spinal Cord Injuries"/ or "spinal cord injury"/ or (((spinal or spine) adj2 (injury or injuries or trauma* or compression)) or cadaver* or volunteer*).ti,ab,kf,kw.) and ("Neck"/ or (neck or cervical).ti,ab,kf,kw.)	Maps to “spinal cord injuries”/ in MESH	21,746
		5	or/1-4 [NECK INJURY]		44,033
		6	exp "Spinal Cord Injuries"/ or exp "Spine”/in		68,479
		7	((spine or spinal or vertebra* or coccyx or intervertebral or lumbar or sacrum or sacral) adj2 (injury or injuries or trauma* or compression or cadaver* or volunteer*)).ti,ab,kf,kw.		72,339
		8	6 or 7 [SPINE INJURY]		99,121
		9	5 or 8 [NECK/SPINE INJURY]		121,909
		10	(immobiliz* or immobilis* or immobile or stabiliz* or stabilis* or restrain* or "trap squeeze" or "trapezius squeeze" or "head squeeze" or "trap grip" or "trapezius grip" or "head grip").ti,ab,kf,kw.		510,245
		11	((reduction or reducing or reduce* or restrain* or decreas* or limit or limiting or restrict*) adj2 (movement or movements or motion or mobility or mobile)).ti,ab,kf,kw.		25,380
		12	(neutral adj2 position*).ti,ab,kf,kw.		3,739
		13	"Movement"/ph or "Head Movements"/ or "head movement"/ or (movement or motion or application).ti,ab,kf,kw.	Maps to “Head Movements”/	1,490,080
		14	"Emergency Medical Services"/mt or "First Aid"/mt		8,071
		15	(out-of-hospital or "out of hospital" or prehospital or pre-hospital or pre-hospitalization or prehospitalization or pre-hospitalisation or prehospitalisation or (before adj2 hospital) or (before adj2 hospitalization) or (before adj2 hospitalisation)).ti,ab,kf,kw.		39,891
		16	(EMS or "emergency medical service" or paramedic* or EMT or "emergency medical technician*" or "first responder*" or ambulance*).ti,ab,kf,kw.		71,163
		17	(bystander* or by-stander* or stranger or strangers or layperson* or lay or public or "first aid" or "in position found").ti,ab,kf,kw.		681,604
		18	"Orthotic Devices"/ or "orthoses"/ or "Braces"/ or "brace"/ or "Restraint, Physical"/mt or (orthotic* or orthosis or orthoses or orthesis or "orthopedic support device*" or "orthopaedic support device*" or collar* or brace* or bracing or restraint*).ti,ab,kf,kw.	Removed or "orthoses"/ or "brace"/ maps to MESH in string	67,389
		19	(or/13-17) and 18		10,385
		20	or/10-12,19 [IMMOBILIZATION/STABILIZATION]		542,306
		21	9 and 20 [(NECK/SPINE INJURY) + (IMMOBILIZATION/STABILIZATION]		8,264
		22	(Animals/ or "Animal Experimentation"/ or "Models, Animal"/ or "Disease Models, Animal"/) not (Humans/ or "Human Experimentation"/)		5,082,526
		23	(exp "animal model"/ or exp "animal experiment"/ or "nonhuman"/ or exp "vertebrate"/) not (exp "human"/ or exp "human experiment"/)	Removed this line of Embase Emtree terms	
		24	21 not (22 or 23) [(NECK/SPINE INJURY) + (IMMOBILIZATION/STABILIZATION), HUMAN]	Removed reference to 23	7795
		25	(comment or letter or "newspaper article" or news or note).pt.	Removed “note”—not MESH	1,910,407
		26	24 not 25 [(NECK/SPINE INJURY) + (IMMOBILIZATION/STABILIZATION), HUMAN, SUBSTANTIVE]		7,750
		27	(Randomized Controlled Trial or Controlled Clinical Trial or Pragmatic Clinical Trial or Equivalence Trial or Clinical Trial, Phase III).pt.		686,909
		28	Randomized Controlled Trial/		591,628
		29	exp Randomized Controlled Trials as Topic/		165,582
		30	Controlled Clinical Trial/		95,284
		31	exp Controlled Clinical Trials as Topic/		171,288
		32	Randomization/	Maps to random allocation in MESH	
		33	Random Allocation/		106,927
		34	Double-Blind Method/		174,998
		35	Double-Blind Studies/	Maps to Double-Blind Method in MESH	
		36	Single-Blind Method/		32,659
		37	Single-Blind Studies/	Maps to Double Blind Method in MESH	
		38	Placebos/		35,926
		39	Control Groups/		1,935
		40	Control Group/	Maps to Control Groups in MESH	
		41	(random* or sham or placebo*).ti,ab,kf,kw.		1,571,208
		42	((singl* or doubl*) adj (blind* or dumm* or mask*)).ti,ab,kf,kw.		195,226
		43	((tripl* or trebl*) adj (blind* or dumm* or mask*)).ti,ab,kf,kw.		1,582
		44	(control* adj3 (study or studies or trial* or group*)).ti,ab,kf,kw.		1,212,594
		45	(nonrandom* or non random* or non-random* or quasi-random* or quasirandom*).ti,ab,kf,kw.		53,310
		46	allocated.ti,ab,kf,kw.		82,868
		47	((open label or open-label) adj5 (study or studies or trial*)).ti,ab,kf,kw.		44,188
		48	((equivalence or superiority or non-inferiority or noninferiority) adj3 (study or studies or trial*)).ti,ab,kf,kw.		11,251
		49	(pragmatic study or pragmatic studies).ti,ab,kf,kw.		589
		50	((pragmatic or practical) adj3 trial*).ti,ab,kf,kw.		5,910
		51	((quasiexperimental or quasi-experimental) adj3 (study or studies or trial*)).ti,ab,kf,kw.		11,963
		52	(phase adj3 (III or "3") adj3 (study or studies or trial*)).ti,ab,kf,kw.		49,970
		53	or/27-52 [RCT/CT]		2,586,451
		54	"Observational Studies as Topic"/ or observational study.pt.		149,623
		55	Cohort Studies/ or Follow-Up Studies/ or Longitudinal Studies/ or Prospective Studies/ or Retrospective Studies/		2,473,790
		56	Cross-Sectional Studies/		464,306
		57	"Evaluation Studies as Topic"/ or evaluation studies.pt.		122,498
		58	Case-Control Studies/		327,429
		59	Comparative Study.pt.		1,912,460
		60	(((evaluation or cohort or cohorts or longitudinal or followup or follow-up or prospective or observational or retrospective or population-based or multidimensional or multi-dimensional or case-control or comparative or cross-sectional or evaluation) adj1 (study or studies)) or "cohort analys*").ti,ab,kf,kw.		1,537,657
		61	or/54-60 [OBSERVATIONAL]		5,193,178
		62	"Epidemiologic Studies"/ or "Cross-Over Studies"/ or "crossover procedure"/	Removed Emtree term	64,305
		63	(((epidemiologic* or intervention or experimental) adj1 (study or studies)) or cross-over or crossover or questionnaire* or survey*).ti,ab,kf,kw.		1,643,942
		64	("before and after" or "interrupted time series").ti,ab,kf,kw.		330,778
		65	"case series".ti,ab,kf,kw.		101,987
		66	or/62-65 [ADDITIONAL STUDIES]		2,047,293
		67	26 and (53 or 61 or 66) [(NECK/SPINE INJURY) + (IMMOBILIZATION/STABILIZATION), HUMAN, SUBSTANTIVE, WITH STUDY FILTERS]		3,317
		68	limit 67 to yr="1999 -Current" [Limit not valid in DARE; records were retained]		2,683
		69	limit 62 to dt=20191001-20230429		403
Ovid MEDLINE(R) ALL <1946 to July 30, 2024>	July 31, 2024	1	exp "Neck Injuries"/ or exp "Cervical Vertebrae"/in		16,530
		2	(exp "Neck"/ or exp "Cervical Vertebrae"/) and (injury or injuries or trauma* or compression).ti,ab,kf,kw.		16,872
		3	(((neck or cervical) adj2 (injury or injuries or trauma* or compression or cadaver* or volunteer*)) or whiplash*).ti,ab,kf,kw.		15,138
		4	("Spinal Cord Injuries"/ or (((spinal or spine) adj2 (injury or injuries or trauma* or compression)) or cadaver* or volunteer*).ti,ab,kf,kw.) and ("Neck"/ or (neck or cervical).ti,ab,kf,kw.)		23,019
		5	1 or 2 or 3 or 4		46,027
		6	exp "Spinal Cord Injuries"/ or exp "Spine"/in		71,065
		7	((spine or spinal or vertebra* or coccyx or intervertebral or lumbar or sacrum or sacral) adj2 (injury or injuries or trauma* or compression or cadaver* or volunteer*)).ti,ab,kf,kw.		77,523
		8	6 or 7		104,632
		9	5 or 8		128,440
		10	(immobiliz* or immobilis* or immobile or stabiliz* or stabilis* or restrain* or "trap squeeze" or "trapezius squeeze" or "head squeeze" or "trap grip" or "trapezius grip" or "head grip").ti,ab,kf,kw.		544,789
		11	((reduction or reducing or reduce* or restrain* or decreas* or limit or limiting or restrict*) adj2 (movement or movements or motion or mobility or mobile)).ti,ab,kf,kw.		27,777
		12	(neutral adj2 position*).ti,ab,kf,kw.		3,959
		13	"Movement"/ph or "Head Movements"/ or (movement or motion or application).ti,ab,kf,kw.		1,630,849
		14	"Emergency Medical Services"/mt or "First Aid"/mt		8,367
		15	(out-of-hospital or "out of hospital" or prehospital or pre-hospital or pre-hospitalization or prehospitalization or pre-hospitalisation or prehospitalisation or (before adj2 hospital) or (before adj2 hospitalization) or (before adj2 hospitalisation)).ti,ab,kf,kw.		43,945
		16	(EMS or "emergency medical service" or paramedic* or EMT or "emergency medical technician*" or "first responder*" or ambulance*).ti,ab,kf,kw.		78,464
		17	(bystander* or by-stander* or stranger or strangers or layperson* or lay or public or "first aid" or "in position found").ti,ab,kf,kw.		758,679
		18	"Orthotic Devices"/ or "Braces"/ or "Restraint, Physical"/mt or (orthotic* or orthosis or orthoses or orthesis or "orthopedic support device*" or "orthopaedic support device*" or collar* or brace* or bracing or restraint*).ti,ab,kf,kw.		71,289
		19	(or/13-17) and 18		11,179
		20	or/10-12,19		579,749
		21	9 and 20		8,615
		22	(Animals/ or "Animal Experimentation"/ or "Models, Animal"/ or "Disease Models, Animal"/) not (Humans/ or "Human Experimentation"/)		5,209,846
		23	21 not 22		8,123
		24	(comment or letter or "newspaper article" or news or study guide).pt.		2,014,778
		25	23 not 24		8,066
		26	(Randomized Controlled Trial or Controlled Clinical Trial or Pragmatic Clinical Trial or Equivalence Trial or Clinical Trial, Phase III).pt.		713,864
		27	Randomized Controlled Trial/		617,974
		28	exp Randomized Controlled Trials as Topic/		176,173
		29	Controlled Clinical Trial/		95,579
		30	exp Controlled Clinical Trials as Topic/		181,981
		31	Random Allocation/		107,462
		32	Double-Blind Method/		179,644
		33	Single-Blind Method/		33,775
		34	Placebos/		35,979
		35	Control Groups/		2,131
		36	(random* or sham or placebo*).ti,ab,kf,kw.		1,705,281
		37	((singl* or doubl*) adj (blind* or dumm* or mask*)).ti,ab,kf,kw.		205,503
		38	((tripl* or trebl*) adj (blind* or dumm* or mask*)).ti,ab,kf,kw.		1,880
		39	(control* adj3 (study or studies or trial* or group*)).ti,ab,kf,kw.		1,315,732
		40	(nonrandom* or non random* or non-random* or quasi-random* or quasirandom*).ti,ab,kf,kw.		58,023
		41	allocated.ti,ab,kf,kw.		90,325
		42	((open label or open-label) adj5 (study or studies or trial*)).ti,ab,kf,kw.		48,610
		43	((equivalence or superiority or non-inferiority or noninferiority) adj3 (study or studies or trial*)).ti,ab,kf,kw.		13,010
		44	(pragmatic study or pragmatic studies).ti,ab,kf,kw.		665
		45	((pragmatic or practical) adj3 trial*).ti,ab,kf,kw.		6,781
		46	((quasiexperimental or quasi-experimental) adj3 (study or studies or trial*)).ti,ab,kf,kw.		14,113
		47	(phase adj3 (III or "3") adj3 (study or studies or trial*)).ti,ab,kf,kw.		54,700
		48	or/26-47		2,776,878
		49	"Observational Studies as Topic"/ or observational study.pt.		168,827
		50	Cohort Studies/ or Follow-Up Studies/ or Longitudinal Studies/ or Prospective Studies/ or Retrospective Studies/		2,632,400
		51	Cross-Sectional Studies/		509,765
		52	"Evaluation Studies as Topic"/ or evaluation studies.pt.		122,502
		53	Case-Control Studies/		336,801
		54	Comparative Study.pt.		1,922,453
		55	(((evaluation or cohort or cohorts or longitudinal or followup or follow-up or prospective or observational or retrospective or population-based or multidimensional or multi-dimensional or case-control or comparative or cross-sectional or evaluation) adj1 (study or studies)) or "cohort analys*").ti,ab,kf,kw.		1,724,649
		56	or/49-55		5,480,664
		57	"Epidemiologic Studies"/ or "Cross-Over Studies"/		66,687
		58	(((epidemiologic* or intervention or experimental) adj1 (study or studies)) or cross-over or crossover or questionnaire* or survey*).ti,ab,kf,kw.		1,789,966
		59	("before and after" or "interrupted time series").ti,ab,kf,kw.		355,385
		60	"case series".ti,ab,kf,kw.		113,449
		61	or/57-60		2,224,753
		62	48 or 56 or 61		8,499,656
		63	25 and 62		3,425
		64	limit 63 to yr="1999 -Current"		2,791
Embase <1974 to 2024 July 30>	July 30, 2024	1	exp neck injury/		16,293
		2	exp cervical vertebra/ and (injury or injuries or trauma* or compression).mp.		2,652
		3	exp neck/ and (injury or injuries or trauma* or compression).mp.		12,487
		4	(((neck or cervical) adj2 (injury or injuries or trauma* or compression or cadaver* or volunteer*)) or whiplash*).mp.		28,761
		5	(exp spinal cord injury/ or (((spinal or spine) adj2 (injury or injuries or trauma* or compression)) or cadaver* or volunteer*).mp.) and ("neck"/ or (neck or cervical).mp.)		43,079
		6	1 or 2 or 3 or 4 or 5		73,827
		7	exp spinal cord injury/		95,192
		8	exp spine/ and (injury or injuries or trauma*).mp.		39,782
		9	((spine or spinal or vertebra* or coccyx or intervertebral or lumbar or sacrum or sacral) adj2 (injury or injuries or trauma* or compression or cadaver* or volunteer*)).mp.		136,717
		10	7 or 8 or 9		163,152
		11	6 or 10		202,300
		12	(immobiliz* or immobilis* or immobile or stabiliz* or stabilis* or restrain* or "trap squeeze" or "trapezius squeeze" or "head squeeze" or "trap grip" or "trapezius grip" or "head grip").mp.		689,631
		13	((reduction or reducing or reduce* or restrain* or decreas* or limit or limiting or restrict*) adj2 (movement or movements or motion or mobility or mobile)).mp.		35,901
		14	(neutral adj2 position*).mp.		5,115
		15	"movement (physiology)"/ or exp head movement/		58,406
		16	(movement or motion or application).mp.		2,128,981
		17	(emergency health service/ or first aid/) and (technique* or procedure* or program or method*).ab.		59,496
		18	(out-of-hospital or "out of hospital" or prehospital or pre-hospital or pre-hospitalization or prehospitalization or pre-hospitalisation or prehospitalisation or (before adj2 hospital) or (before adj2 hospitalization) or (before adj2 hospitalisation)).mp.		70,382
		19	(EMS or "emergency medical service" or paramedic* or EMT or "emergency medical technician*" or "first responder*" or ambulance*).mp.		133,111
		20	(bystander* or by-stander* or stranger or strangers or layperson* or lay or public or "first aid" or "in position found").mp.	[mp=title, abstract, heading word, drug trade name, original title, device manufacturer, drug manufacturer, device trade name, keyword heading word, floating subheading word, candidate term word]	1,166,595
		21	or/15-20		3,441,735
		22	"physical restraint"/ and (technique* or procedure* or program or method*).ab.		666
		23	"orthoses"/ or "brace"/		18,022
		24	(orthotic* or orthosis or orthoses or orthesis or "orthopedic support device*" or "orthopaedic support device*" or collar* or brace* or bracing or restraint*).mp.		108,943
		25	22 or 23 or 24		108,943
		26	21 and 25		20,360
		27	12 or 13 or 14 or 26		739,135
		28	11 and 27		15,511
		29	(Randomized controlled trial/ or Controlled clinical study/ or random*.ti,ab. or randomization/ or intermethod comparison/ or placebo.ti,ab. or (compare or compared or comparison).ti. or ((evaluated or evaluate or evaluating or assessed or assess) and (compare or compared or comparing or comparison)).ab. or (open adj label).ti,ab. or ((double or single or doubly or singly) adj (blind or blinded or blindly)).ti,ab. or double blind procedure/ or parallel group*1.ti,ab. or (crossover or cross over).ti,ab. or ((assign* or match or matched or allocation) adj5 (alternate or group*1 or intervention*1 or patient*1 or subject*1 or participant*1)).ti,ab. or (assigned or allocated).ti,ab. or (controlled adj7 (study or design or trial)).ti,ab. or (volunteer or volunteers).ti,ab. or human experiment/ or trial.ti.) not (((random* adj sampl* adj7 ("cross section*" or questionnaire*1 or survey* or database*1)).ti,ab. not (comparative study/ or controlled study/ or randomi?ed controlled.ti,ab. or randomly assigned.ti,ab.)) or (Cross-sectional study/ not (randomized controlled trial/ or controlled clinical study/ or controlled study/ or randomi?ed controlled.ti,ab. or control group*1.ti,ab.)) or (((case adj control*) and random*) not randomi?ed controlled).ti,ab. or (Systematic review not (trial or study)).ti. or (nonrandom* not random*).ti,ab. or "Random field*".ti,ab. or (random cluster adj3 sampl*).ti,ab. or ((review.ab. and review.pt.) not trial.ti.) or ("we searched".ab. and (review.ti. or review.pt.)) or "update review".ab. or (databases adj4 searched).ab. or ((rat or rats or mouse or mice or swine or porcine or murine or sheep or lambs or pigs or piglets or rabbit or rabbits or cat or cats or dog or dogs or cattle or bovine or monkey or monkeys or trout or marmoset*1).ti. and animal experiment/) or (Animal experiment/ not (human experiment/ or human/)))		5,893,245
		30	observational study/		385,605
		31	cohort analysis/		1,201,054
		32	longitudinal study/		218,772
		33	prospective study/		933,950
		34	retrospective study/		1,663,137
		35	clinical study/		167,400
		36	(((evaluation or cohort or cohorts or longitudinal or followup or follow-up or prospective or observational or retrospective or population-based or multidimensional or multi-dimensional or case-control or comparative or cross-sectional or evaluation) adj1 (study or studies)) or "cohort analys*").mp.		5,524,994
		37	(((epidemiologic* or intervention or experimental) adj1 (study or studies)) or cross-over or crossover or questionnaire* or survey*).mp.		3,343,548
		38	("before and after" or "interrupted time series").mp.		497,881
		39	"case series".mp.		161,687
		40	or/29-39		12,139,904
		41	28 and 40		6,090
		42	(comment or letter or "newspaper article" or news or note or conference).pt.		8,323,644
		43	41 not 42		4,720
		44	limit 43 to yr="1999 -Current"		4,207
CINAHL Plus with Full Text (1936 - Present) via EBSCOhost	April 30, 2024	S1	(MH "Neck Injuries+")		3,866
		S2	(MH "Cervical Vertebrae+/IN")		2,438
		S3	(MH "Neck+")		7,754
		S4	(MH "Cervical Vertebrae+)		14,124
		S5	(injury or injuries or trauma* or compression)		486,889
		S6	S3 OR S4		21,336
		S7	S5 AND S6		6,549
		S8	(((neck or cervical) N2 (injury or injuries or trauma* or compression or cadaver* or volunteer*)) or whiplash*)		9,901
		S9	(MH "Spinal Cord Injuries+")		23,831
		S10	(((spinal or spine) N2 (injury or injuries or trauma* or compression)) or cadaver* or volunteer*)		110,095
		S11	S9 OR S10		110,120
		S12	(MH "Neck") or (neck or cervical)		134,630
		S13	S11 AND S12		8,419
		S14	S1 OR S2 OR S7 OR S8 OR S13		15,892
		S15	(MH "Spine+/IN") or (MH "Spinal Cord Injuries+")		27,516
		S16	((spine or spinal or vertebra* or coccyx or intervertebral or lumbar or sacrum or sacral) N2 (injury or injuries or trauma* or compression or cadaver* or volunteer*))		39,134
		S17	S14 OR S15 OR S16		47,740
		S18	(immobiliz* or immobilis* or immobile or stabiliz* or stabilis* or restrain* or "trap squeeze" or "trapezius squeeze" or "head squeeze" or "trap grip" or "trapezius grip" or "head grip")		52,313
		S19	((reduction or reducing or reduce* or restrain* or decreas* or limit or limiting or restrict*) N2 (movement or movements or motion or mobility or mobile))		8,665
		S20	(neutral N2 position*)		1,466
		S21	S18 OR S19 OR S20		61,532
		S22	(MH "Movement+/PH")		21,394
		S23	(movement or motion or application)		349,037
		S24	(MH "Emergency Medical Services/MT")		2,181
		S25	(MH "First Aid/MT")		186
		S26	(out-of-hospital or "out of hospital" or prehospital or pre-hospital or pre-hospitalization or prehospitalization or pre-hospitalisation or prehospitalisation or (before N2 hospital) or (before N2 hospitalization) or (before N2 hospitalisation))		31,727
		S27	(EMS or "emergency medical service" or paramedic* or EMT or "emergency medical technician*" or "first responder*" or ambulance*)		37,161
		S28	(bystander* or by-stander* or stranger or strangers or layperson* or lay or public or "first aid" or "in position found")		354,668
		S29	S22 OR S23 OR S24 OR S25 OR S26 OR S27 OR S28		749,053
		S30	(MH "Orthoses+")		11,485
		S31	(MH "Restraint, Physical/MT")		206
		S32	(MH "Cervical Collars")		207
		S33	(orthotic* or orthosis or orthoses or orthesis or "orthopedic support device*" or "orthopaedic support device*" or collar* or brace* or bracing or restraint*)		31,966
		S34	S30 OR S31 OR S32 OR S33		32,481
		S35	S29 AND S34		6,610
		S36	S21 OR S35		65,688
		S37	S17 AND S36		3,377
		S38	( MH ( randomized controlled trials OR double‐blind studies OR single‐blind studies OR random assignment OR pretest‐posttest design OR cluster sample ) OR TI ( randomised OR randomized ) OR AB random* OR TI trial OR ( (MH (sample size) AND AB (assigned OR allocated OR control)) ) OR MH ( placebos OR crossover design OR comparative studies ) OR AB ( (control W5 group) OR (cluster W3 RCT) OR PT (randomized controlled trial)) ) NOT ( ( MH animals+ OR MH (animal studies) OR TI (animal model*) ) NOT MH (human) )		1,013,942
		S39	(((evaluation or cohort or cohorts or longitudinal or followup or follow-up or prospective or observational or retrospective or population-based or multidimensional or multi-dimensional or case-control or comparative or cross-sectional or evaluation) N1 (study or studies)) or "cohort analys*")		1,424,107
		S40	(((epidemiologic* or intervention or experimental) N1 (study or studies)) or cross-over or crossover or questionnaire* or survey*)		1,012,886
		S41	("before and after" or "interrupted time series")		79,309
		S42	"case series"		32,406
		S43	S38 OR S39 OR S40 OR S41 OR S42		2,393,311
		S44	S37 AND S43		1,368
		S45	S37 AND S43 "Limiters - Publication Date: 19990101-20241231 Search modes - Find all my search terms"		1,227
		S46	S37 AND S43 "Limiters - Scholarly (Peer Reviewed) Journals; Publication Date: 19990101-20241231 Search modes - Find all my search terms"		1,206
